# A Guide to the Practical Study of Diseases of the Eye; with an Outline of Their Medical and Operative Treatment

**Published:** 1866-10

**Authors:** 


A Guide to the Practical Study of Diseases of the Eye; With an Outline of their Medical and Operative Treatment. By James Dixon, F.R.C.S., Surgeon to the Royal Ophthalmic Hospital, Moorfields, London. From the third London edition. Philadelphia: Lindsay & Blakiston. 1866.

This is a neat little volume of 400 pages. The former editions of the work have been long enough before the public to be familiar to the reading part of the profession. It is concise, plain, and practical; just such a manual on diseases of the eye as the student and practitioner will find convenient and useful. For sale by W. B. Keen & Co., 148 Lake Street. Price $2.50.

